# Cognitive function in individuals with and without painful and painless diabetic polyneuropathy—A cross‐sectional study in type 1 diabetes

**DOI:** 10.1002/edm2.420

**Published:** 2023-04-18

**Authors:** Suganthiya S. Croosu, Mimoza Gjela, Johan Røikjer, Tine M. Hansen, Carsten D. Mørch, Jens B. Frøkjær, Niels Ejskjaer

**Affiliations:** ^1^ Department of Radiology Aalborg University Hospital Hobrovej 18‐22 9000 Aalborg Denmark; ^2^ Department of Endocrinology Aalborg University Hospital Mølleparkvej 4 9000 Aalborg Denmark; ^3^ Steno Diabetes Center North Denmark Aalborg University Hospital Mølleparkvej 4 9000 Aalborg Denmark; ^4^ Department of Clinical Medicine Aalborg University Søndre Skovvej 15 9000 Aalborg Denmark; ^5^ Center for Neuroplasticity and Pain (CNAP), SMI, Department of Health Science and Technology Aalborg University Fredrik Bajers Vej 7D 9220 Aalborg Denmark

**Keywords:** ACE‐III, cognitive function, diabetic peripheral neuropathy, memory, N‐back task, neuropathic pain, type 1 diabetes

## Abstract

**Introduction:**

Previous studies suggest that cognitive impairment is more prevalent in individuals with painful and painless diabetic peripheral neuropathy (DPN). However, the current evidence is not well described. This study investigated cognitive function in adults with type 1 diabetes mellitus (T1DM) and the association to painful/painless DPN and clinical parameters.

**Methods:**

This cross‐sectional, observational, case–control study included 58 participants with T1DM, sub‐grouped into 20 participants with T1DM and painful DPN, 19 participants with T1DM and painless DPN, 19 participants with T1DM without DPN, and 20 healthy controls were included. The groups were matched for sex and age. The participants performed Addenbrooke's examination III (ACE‐III), which assesses attention, memory, verbal fluency, language and visuospatial skills. Working memory was evaluated using an N‐back task. Cognitive scores were compared between the groups and correlated to age, diabetes duration, HbA1c and nerve conduction measurements.

**Results:**

Compared to healthy controls, T1DM participants showed lower total ACE‐III (*p* = .028), memory (*p* = .013) and language scores (*p* = .028), together with longer reaction times in the N‐back task (*p* = .041). Subgroup analyses demonstrated lower memory scores in those with painless DPN compared with healthy controls (*p* = .013). No differences were observed between the three T1DM subgroups. Cognitive scores and clinical parameters were not associated.

**Conclusions:**

This study supports the notion of cognitive alterations in T1DM and indicates that cognitive function is altered in T1DM regardless of underlying neuropathic complications. The memory domain appears altered in T1DM, particularly in those with painless DPN. Further studies are needed to verify the findings.

## Graphical abstract

This study investigated cognitive function in adults with type 1 diabetes mellitus (T1DM) and the association to painful/painless DPN and clinical parameters. The study supports the notion of cognitive alterations in T1DM and indicates that cognitive function is altered in T1DM regardless of underlying neuropathic complications. The memory domain appears altered in T1DM, particularly in those with painless DPN. Further studies are needed to verify the findings.

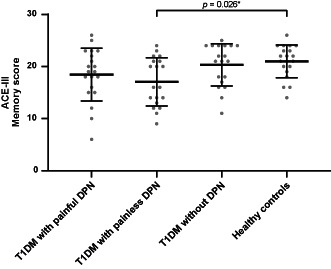

## 1 INTRODUCTION

Cognitive function has been suggested to be mildly to moderately affected in individuals with type 1 diabetes mellitus (T1DM).^1^, ^2^, ^3^ Clinically significant cognitive impairment in 28% of a cohort with middle‐aged T1DM participants with childhood‐onset diabetes was found, while a prevalence of 5% was observed in healthy controls.^4^ The underlying mechanisms are unknown, but it has been suggested that cognitive impairments in T1DM may be attributed to several diabetes‐related factors including higher HbA1c, retinopathy and diabetic peripheral neuropathy (DPN).^4^, ^5^ DPN is the most common complication of T1DM,^6^ affecting up to 50% of all diabetic individuals.^7^, ^8^ It presents with loss of sensitivity in lower extremities^6^ and can have severe consequences due to an elevated risk of developing foot ulcers, which may lead to amputations and premature death.^9^, ^10^

In one study, individuals with T1DM and mild or clinically relevant cognitive impairment had a higher DPN prevalence than T1DM participants who presented with normal cognitive function.^4^ Another study demonstrated a correlation between decreased nerve conduction velocity and decreased cognitive function in individuals with T1DM, indicating a possible link between changes in the peripheral and central nervous systems.^5^ Also, specific cognitive domains have been reported affected in T1DM with DPN including psychomotor speed and attention.^11^

Another factor associated with cognitive impairment is chronic pain conditions, where problems with concentration, memory, processing and attention are prevalent.^12^, ^13^ A common cause of chronic pain is peripheral neuropathic pain, a condition that has shown changes in the neural pathways involved in cognition.^9^, ^14^ Peripheral neuropathic pain is experienced by 15–25% of individuals with diabetes and DPN in their lower extremities and is known as painful DPN.^9^ Considering that diabetes, painless DPN and painful DPN appear to be related to cognitive impairment a combination of these may increase the risk of cognitive impairment. Limited attention has been placed on understanding the underlying mechanisms of T1DM‐related cognitive impairment and the impact of factors such as painful and painless DPN on cognitive function. This is necessary to prevent further cognitive decline and offer appropriate supportive measures. The present study hypothesized that individuals with T1DM in general would present lower cognitive scores compared to healthy controls and that painful and painless DPN contribute to cognitive impairment. Firstly, the current explorative study aimed to investigate cognitive function in a mixed cohort of adults with T1DM both with and without painful/painless DPN compared to healthy controls. A limited number of studies have investigated the impact of DPN on cognitive function. To our knowledge, no studies have examined the difference in cognitive function in different phenotypes of DPN in T1DM. Hence, the study also aimed to investigate cognitive function in subgroups of individuals with T1DM and painful DPN, T1DM and painless DPN, T1DM without DPN, and healthy controls. Furthermore, associations between impaired cognitive function in T1DM and several disease characteristics have been observed in other studies.^3^, ^15^, ^16^ However, their impact on cognitive function has still not been fully elucidated. The final aim was therefore to explore associations between cognitive scores and clinical parameters, including age, diabetes duration and HbA1c. Parameters reflecting DPN such as nerve conduction measurements were also included.

## 2 MATERIALS AND METHODS

### 2.1 Study design and participants

This was a cross‐sectional, observational, case–control study performed at the Department of Endocrinology, Steno Diabetes Center North Denmark and Department of Radiology, Aalborg University Hospital, Denmark. The study was part of a larger clinical study named MEDON (Methods of Early Detection of diabetic peripheral Neuropathy) in which the primary outcome was to examine alterations of the peripheral small nerve fibres. Hence, the aim of the present cognitive sub‐study was pursued in an explorative manner. The cohort and methods are described in detail elsewhere.^17^, ^18^, ^19^, ^20^ Participants were recruited from the outpatient clinic at the Department of Endocrinology, Aalborg University Hospital, between August 2019 and April 2021. The current cognitive study included 58 participants diagnosed with T1DM and 20 participants without diabetes and neuropathic complications designated as healthy controls. The T1DM group consisted of three subgroups: 20 participants with T1DM and painful DPN, 19 participants with T1DM and confirmed painless DPN, and 19 participants with T1DM without painful or painless DPN. See section ‘Subgrouping and clinical parameters’.

Each participant in each subgroup was matched for sex and age (±2 years) to a participant in the other three subgroups. The inclusion criteria for all participants were: Men and women between 18 and 70 years. The diabetes group were included if they were diagnosed with T1DM with further specific criteria applied for the different subgroups of diabetes. Those with T1DM and painful DPN were included if they scored 4 or higher on Douleur Neuropathique 4 Questions (DN4) questionnaire, those with T1DM and painless DPN were included if they had an abnormal vibration perception threshold on the toe (above 25‐volt, biothesiometry test), and those with T1DM without DPN were included if they had a normal vibration perception threshold (below 25‐volt). Healthy controls were included if they were not diagnosed with diabetes nor presented with neuropathic complications, including abnormal peripheral vibration perception threshold.

Exclusion criteria for participants with T1DM and healthy controls included previous or current alcohol and/or drug abuse, presence of chronic viral infection, known neural damage or disease in the neural system or critical ischemia of the lower extremities; severe skin disease; pregnancy, active cancer‐disease and previous chemotherapy or consumption of experimental medicine. All participants provided informed written consent before trial enrollment. The study was conducted according to the Declaration of Helsinki. Ethical approvals were granted by The North Denmark Region Committee on Health Research Ethics (N‐20190003) and registered with clinicaltrials.gov (NCT04078516).

### 2.2 Subgrouping and clinical parameters

Painful DPN was clinically confirmed by two independent medical doctors and supported by the self‐reporting DN4 questionnaire. A score of 4 or above was considered abnormal, and these participants were classified as having painful DPN.^21^

Painless DPN was confirmed according to the Toronto consensus.^7^ Thus, those with abnormal vibration perception threshold (above 25 V) and the presence of an abnormal nerve conduction study (NCS)^7^ were included in the group with T1DM and painless DPN. The vibration sensation test was performed on the participant's great toe. The NCS was performed on the right leg on standardized skin temperature at the Department of Neurophysiology, Aalborg University Hospital, according to clinical guidelines. Nerve conduction velocity, amplitude and latency were evaluated on the sural nerve to confirm painless DPN.

Other obtained clinical parameters included retinopathy status, which was diagnosed in accordance with the local clinical standards of Department of Ophthalmology, Aalborg University Hospital, Denmark, where the minimum diagnosis criteria for retinopathy were retinal microaneurysms.^22^ Due to the risk of excessive glucose levels in T1DM participants, which may affect cognitive performance, actual glucose levels were obtained before performing the cognitive task. Furthermore, blood samples were taken from all participants to measure HbA1c.

### 2.3 Assessment of cognitive function

#### 2.3.1 Addenbrooke's examination

The participants performed Addenbrooke's Cognitive Examination‐III (ACE‐III), a validated cognitive examination that assesses the following five cognitive domains: attention (18 points), memory (26 points), verbal fluency (14 points), language (26 points) and visuospatial abilities (16 points).^23^ Attention was assessed by inquiring about the date, recalling three words and performing serial subtraction. Memory was tested by recalling the three previously repeated words, a fictional name and address, and historical facts. Fluency was evaluated by participant generating words starting with a specific letter and naming animals. Language was tested through completing physical commands using a pencil and paper, writing sentences, repeating polysyllabic words and proverbs, identifying objects in line drawings, and reading irregular words. Visuospatial abilities were assessed by copying diagrams, drawing a clock face, counting dots and recognizing fragmented letters. Further details on ACE‐III test can be found elsewhere.^23^, ^24^ The total possible score is 100, and higher scores indicate better cognitive function. Several studies have proposed two cut‐off scores of 82 and 88 points to screen for cognitive impairment.^25^ The lower threshold was used for this study to ensure the presence of cognitive impairment.

#### 2.3.2 N‐back task

The participants performed a visual N‐back task, which tests a person's ability to temporary storage, update, manipulation of remembered information and respond to a stimulus. In other words, the task tests the cognitive domains, working memory and psychomotor speed.^26^ During the task, a sequence of letters was presented one by one. For each letter, the participant had to decide if the letter was presented *N* letters previously. The higher number of *N*, the more difficult the task is. Before performing the task, the participants underwent a practice session consisting of a shorter version of the N‐back task to become familiar with the task. This study used three blocks in random order: 0‐back, 1‐back and 2‐back. During the 0‐back block, participants were instructed to click on a button every time a predetermined letter appeared on the screen (target letter) and ignore any other letters (distraction letters). During the 1‐back block, participants were instructed to click on the button every time the same letter was repeated. Lastly, in the 2‐back block, participants had to click on the button when the presented letter had appeared two letters earlier in the sequence. Each block lasted 122 s and included 50 letter stimuli, of which 10 were targets and 40 were distractors. Letters were presented for 0.5 s, and the interstimulus interval lasted 1.5 s.

Three parameters were obtained for each participant based on the N‐back tasks, which included a discrimination index d‐prime (*d*′), reaction times (RT) and weighted RT. The *d*′ was calculated based on hits (correct clicks on the button when target letters appeared), misses (missed clicks on the button when target letters appeared), false alarms (clicked on the button on distractor letters) and correct negatives (no clicks on distractor letters) using the following formula: $d^{\prime }=Z_{\text{Hits}}–Z_{\mathrm{FA}}$.^27^ *Z*_Hits_ represents the transformed hit rate and was calculated by transforming the following value: $\text{hits}/\left(\text{hits}+\text{misses}\right)$. *Z*_FA_ represents the transformed false alarms rate and was calculated by transforming the following value: false alarms/(false alarms+correct negative) (Haatveit et al., 2010). *d*′ reflects the sensitivity of the participants to discriminate target letters from distraction letters. Thus, a high *d*′ indicates that the target is easily detected.^27^ The RT reflect the psychomotor performance, thus how quickly the participants react to the visual stimuli in the N‐back task with a motor activity (pressing the button). However, the raw RT value is associated with the speed‐accuracy trade‐off problem, meaning that decisions are made slowly with high accuracy or fast with a high error rate.^26^, ^28^, ^29^ To overcome this problem, the RT was adjusted for accuracy generating weighted RTs (RTW) using the following formula: $\mathrm{RTW}=\mathrm{RT}+\left(\mathrm{RT}\;\left(1-\text{Accuracy}\right)\right)$, where accuracy was hits/targets.^26^

### 2.4 Statistical analyses

Demographical, clinical, ACE‐III and N‐back data were tested for normal distribution using the Shapiro–Wilk test and Q–Q plots. Depending on the distribution, independent t‐tests or Mann–Whitney U tests were performed to compare the T1DM group and healthy controls. Sex, retinopathy status and medications were compared using χ^2^‐or fisher's exact tests. For the comparisons across subgroups, one‐way ANOVAs and Kruskal‐Wallis tests were used. Post‐hoc tests with Bonferroni corrections for multiple comparisons were used. Spearman's correlations were performed to examine associations between cognitive scores and clinical parameters. Cognitive scores which showed significant differences between the tested groups were chosen for the correlation analysis. Data are presented as mean ± standard deviation or median (interquartile range). *p* < .05 was considered significant. Statistical analyses were performed using IBM SPSS Statistics for Windows, version 27.0. IBM Corp. Released 2020. Armonk, NY.

## 3 RESULTS

All 58 participants included in the current cognitive study completed the cognitive tasks and were included in the reported results. Due to impaired vision, one participant did not complete parts of the cognitive tasks which required visual attention therefore this participant was only included in parts of the analysis. Tables 1 and 2 summarize the demographic and clinical data together with peripheral measurements, neuropathic pain assessments and cognitive scores for the overall T1DM group versus healthy controls and the subgroups versus healthy controls respectively.

**TABLE 1:** Demographic, clinical characteristics and cognitive scores for the T1DM participants and healthy controls. Data are presented as mean ± SD unless otherwise stated.

	T1DM	Healthy controls	*p*‐Value
Demographic and clinical characterization			
*N*	58	20	
Sex (Male/Female)	29/29	10/10	.946
Age (years)^b^	51.0 (44.0; 57.0)	50.5 (44.0; 59.3)	.954
BMI (kg/m^2^)^b^	27.2 (24.2;30.3)	24.3 (23.0; 28.6)	.056
Age of onset (years)^b^	19.0 (10.8; 30.3)		
Duration (years)	29.5 ± 12.1		
Glucose (mg/dL)	10.6 ± 3.6		
HbA1c (mmol/mol)	68.8 ± 11.1	33.5 ± 3.3	<.001^a^
Rethinopathy (yes/no (%))	48/10 (82.8/17.2%)	0/20 (0.0/100.0%)	<.001^a^
Peripheral nerve measurements			
Sural amplitude (μV)^b^	2.1 (0.0; 4.6)	10.3 (5.5; 13.6)	<.001^a^
Sural velocity (m/s)^b^	38.5 (0.0; 46.3)	54.5 (47.5; 57.8)	<.001^a^
Neuropathic pain assessments			
DN4 score^b^	0.5 (0.0; 4.0)	0.0 (0.0; 0.0)	<.001^a^
NRS peak pain last 4 weeks^b^	0.0 (0.0; 7.0)	0.0 (0.0; 0.0)	<.001^a^
NRS average pain last 4 weeks^b^	0.0 (0.0; 5.0)	0.0 (0.0; 0.0)	.001^a^
Medications (*n*(%))			
Medications for neuropathic pain	15 (25.9)	0 (0)	.009^a^
Other CNS‐acting medications	9 (15.5)	0 (0)	.102
Cognitive scores			
ACE‐III			
Total^b^	90.0 (83.0; 94.0)^c^	94.0 (87.5; 95)	.028^a^
Attention^b^	18.0 (17.0; 18.0)	18.0 (17.3;18.0)	.426
Memory	18.6 ± 4.7	21.0 ± 3.1	.013^a^
Verbal fluency^b^	12.0 (11.0; 13.0)	12.0 (11.3;14.0)	.326
Language^b^	25.0 (24.0; 25.0)^c^	25.0 (25.0;26.0)	.028^a^
Visuospatial^b^	16.0 (15.0; 16.0)^c^	16.0 (15.3;16.0)	.237
N‐back, *d*′			
0‐back^b^	3.9 (3.9; 3.9)^c^	3.9 (3.9; 3.9)	.581
1‐back^b^	3.9 (3.5; 3.9)^c^	3.9 (3.7; 3.9)	.283
2‐back	1.9 ± 0.7^c^	2.3 ± 0.6	.063
N‐back, RT (ms)			
0‐back^b^	447 (417; 477)^c^	422 (397; 438)	.032^a^
1‐back^b^	533 (458; 617)^c^	500 (447; 559)	.236
2‐back^b^	668 (601; 765)^c^	645 (599; 749)	.577
N‐back, RTW (ms)			
0‐back^b^	447 (417; 479)^c^	422 (401; 438)	.041^a^
1‐back^b^	551 (464; 644)^c^	503 (462; 563)	.197
2‐back^b^	873 (794; 1125)^c^	813 (696; 990)	.128

Abbreviations: ACE‐III, Addenbrooke's Cognitive Examination; BMI, body mass index; d’, the discrimination index d‐prime; DN4, Douleur Neuropathique 4 Questions; HbA1c, haemoglobin A1c; NRS, Numeric pain rating scale; RT, reaction time; RTW, weighted reaction times; T1DM, type 1 diabetes mellitus.

^a^ Represents a statistically significant difference between the groups.

^b^ Presented as median (interquartile range).

^c^ *n* = 57 (One participant with impaired vision was excluded from the visual tasks due to difficulties).

**TABLE 2:** Demographic, clinical characteristics, and cognitive scores for T1DM with painful DPN, T1DM with painless DPN, T1DM without DPN, and healthy controls.

	T1DM with painful DPN	T1DM with painless DPN	T1DM without DPN	Healthy controls	*p*‐Value
Demographic and clinical characterization					
*N*	20	19	19	20	
Sex (Male/Female)	10/10	10/9	10/9	10/10	.984
Age (years)^b^	50.5 (43.3; 57.0)	52.0 (45.0; 60.0)	49.0 (44.0; 57.0)	50.5 (44.0; 59.3)	.939
BMI (kg/m^2^)^b^	27.2 (24.7; 30.8)	27.7 (24.2; 30.3)	27.0 (24.1; 30.3)	24.3 (23.0; 28.6)	.299
Age of onset (years)^b^	17.5 (9.3; 27.0)	19.0 (11.0;25.0)	21.0 (11.0;39.0)		.527
Duration (years)	30.5 ± 13.7	33.7 ± 8.1	24.4 ± 12.2		.053
Glucose (mg/dL)	10.8 ± 3.4	11.0 ± 3.8	10.1 ± 3.7		.736
HbA1c (mmol/mol)	69.1 ± 11.4	73.5 ± 10.4	63.9 ± 9.9	33.5 ± 3.3	<.001^a^
Retinopathy (yes/no (%))	19/1 (95.0/5.0%)	17/2 (89.5/10.5%)	12/7 (63.2/36.8%)	0/20 (0.0(100.0%)	<.001^a^
Peripheral nerve measurements					
Sural amplitude (μV)	0.4 (0.0; 3.3)	0.0 (0.0; 3.3)	5.4 (2.9; 7.9)	10.3 (5.5; 13.6)	<.001^a^
Sural velocity (m/s)	13.5 (0.0; 39.8)	0.0 (0.0; 38.0)	48.0 (45.0; 50.0)	54.5 (47.5; 57.8)	<.001^a^
Neuropathic pain assessments					
DN4 score^b^	5.0 (4.0; 6.0)	0.0 (0.0; 2.0)	0.0 (0.0; 0.0)	0.0 (0.0; 0.0)	<.001^a^
NRS peak pain last 4 weeks^b^	8.0 (6.0; 9.0)	0.0 (0.0; 1.0)	0.0 (0.0; 0.0)	0.0 (0.0; 0.0)	<.001^a^
NRS average pain last 4 weeks^b^	5.0 (4.0; 7.75)	0.0 (0.0; 0.0)	0.0 (0.0; 0.0)	0.0 (0.0; 0.0)	<.001^a^
Medications (*n*(%))					
Medications for neuropathic pain	15 (75.0)	0(0)	0(0)	0(0)	<.001^a^
Other CNS‐acting medications	4(20.0)	5(26.3)	0(0)	0(0)	.006^c^
Cognitive scores					
ACE‐III					
Total^b^	88.5 (84.3; 92.5)	85.5 (80.5; 92.5)^d^	92.0 (84.0; 95.0)	94.0 (87.5; 95.0)	.060
Attention^b^	18.0 (17.0;18.0)	18.0 (17.0;18.0)	18.0 (17.0;18.0)	18.0 (17.3;18.0)	.867
Memory	18.5 ± 5.1	17.1 ± 4.6	20.3 ± 4.1	21.0 ± 3.1	.022^a^
Verbal fluency^b^	12.0 (11.0;13.0)	12.0 (11.0;13.0)	12.0 (11.0;13.0)	12.0 (11.3;14.0)	.782
Language^b^	24.5 (24.0;25.0)	24.5 (23.0; 25.0)^d^	25.0 (24.0;26.0)	25.0 (25.0;26.0)	.111
Visuospatial skills^b^	16.0 (15.0;16.0)	16.0 (13.8;16.0)^d^	16.0 (15.0;16.0)	16.0 (15.3;16.0)	.362
N‐back, *d*′					
0‐back^b^	3.9 (3.7; 3.9)	3.9 (3,9; 3.9)^d^	3.9 (3.9; 3.9)	3.9 (3.9; 3.9)	.081
1‐back^b^	3.8 (3.5; 3.9)	3.9 (3.6; 3.9)^d^	3.9 (3.5; 3.9)	3.9 (3.7; 3.9)	.448
2‐back^b^	1.9 ± 0.8	1.8 ± 0.6^d^	2.1 ± 0.7	2.3 ± 0.6	.116
N‐back, RT (ms)					
0‐back^b^	452 (418; 486)	447 (421; 484)^d^	434 (395; 465)	422 (397; 438)	.158
1‐back^b^	542 (470; 607)	559 (475; 626)^d^	494 (448; 633)	500 (447; 559)	.493
2‐back^b^	714 (562; 752)	653 (547; 721)^d^	687 (617; 805)	645 (599; 749)	.594
N‐back, RTW (ms)					
0‐back^b^	453 (418; 494)	447 (421; 484)^d^	434 (395; 465)	422 (401; 438)	.181
1‐back^b^	557 (486; 667)	587 (475; 646)^d^	506 (448; 645)	503 (462; 563)	.516
2‐back^b^	912 (778; 1127)	872 (774; 1052)^d^	936 (800; 1234)	813 (696; 990)	.462

*Note*: Data are presented as mean ± standard deviation unless otherwise stated.

Abbreviations: ACE‐III, Addenbrooke's Cognitive Examination; BMI, body mass index; CNS, central nervous system; d’, the discrimination index d‐prime; DN4, Douleur Neuropathique 4 Questions; DPN, diabetic peripheral neuropathy; HbA1c, haemoglobin A1c; NRS, Numeric pain rating scale; RT, reaction time; RTW, weighted reaction times; T1DM, type 1 diabetes mellitus.

^a^ Represents an overall statistically significant difference between the groups.

^b^ Presented as median (interquartile range).

^c^ No pairwise statistical significance.

^d^ *n* = 18 (One participant with impaired vision was excluded from the visual tasks due to difficulties).

### 3.1 Demographics

Overall, there were no differences when comparing sex, age and body mass index (BMI) between the T1DM group and the control group (all *p* > .056). As expected, healthy controls had a lower HbA1c value compared to T1DM participants (*p* < .001). Also, the T1DM group demonstrated a higher prevalence of retinopathy and poorer nerve conduction measurements as well as higher pain scores (all *p* ≤ .001). Higher use of neuropathic pain medications was observed in the T1DM group (*p* < .009). See Table 1.

There were no differences when comparing sex, age and BMI between the four subgroups (all *p* > .299). The diabetes subgroups did not differ in regard to the age of diabetes onset, diabetes duration and glucose level (all *p* > .053). Healthy controls had a lower HbA1c concentration compared to the three T1DM subgroups (all *p* < .001). Additionally, the participants with T1DM and painless DPN had higher HbA1c concentrations than those with T1DM without DPN (*p* = .012). All three T1DM subgroups had higher prevalences of retinopathy compared to the healthy controls (*p* < .001). The participants with T1DM and painful DPN and those with T1DM and painless DPN had significantly poorer peripheral nerve conduction measurements compared to the participants with T1DM without DPN and the healthy controls (all *p* < .019). Moreover, the pain score and use of neuropathic pain medications were higher in the group with painful DPN compared to the other three groups (all *p* < .001). See Table 2.

### 3.2 Cognitive alterations in the overall type 1 diabetes group

**FIGURE 1:**
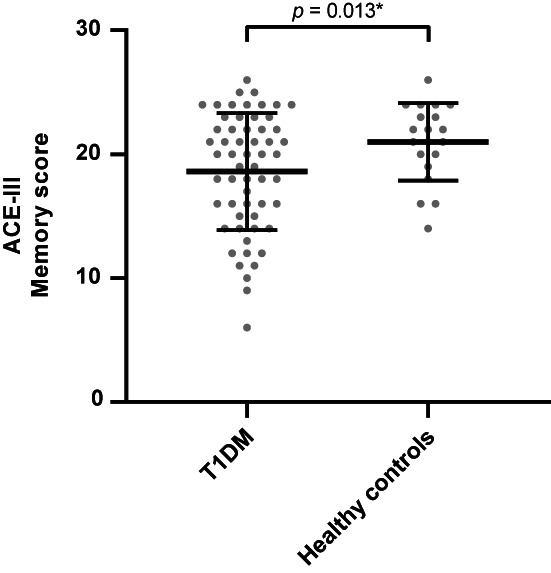
The memory score from ACE‐III for the group with T1DM and the healthy controls. The middle lines demonstrate the mean, and the error lines demonstrate the standard deviation. *Represents a statistically significant difference between the indicated groups. ACE‐III, Addenbrooke's Cognitive Examination; T1DM, type 1 diabetes mellitus.

Overall, participants with T1DM demonstrated a lower total ACE‐III score compared to healthy controls (*p* = .028). Moreover, the memory and language scores were lower in the T1DM group compared to healthy controls (all *p* < .028) (Figure 1). No differences were observed for the other cognitive domains, including attention, verbal fluency and visual and spatial skills (all *p* > .237). Twenty‐one percent of the participants with T1DM and 5% of the healthy controls had a total score below 82. See Table 1.

In the N‐back task, the participants with T1DM demonstrated longer RTs and RTWs during the 0‐back block compared to healthy controls (*p* < .041). However, no differences in RTWs were observed during 1‐back and 2‐back blocks (all *p* > .128, see Table 1). The RTWs increased as the memory load of the N‐back task increased for both the T1DM group and the control group (*p* < .001). The index d’ did not differ between the two groups (all *p* > .063).

### 3.3 Cognitive alterations in subgroups of type 1 diabetes

**FIGURE 2:**
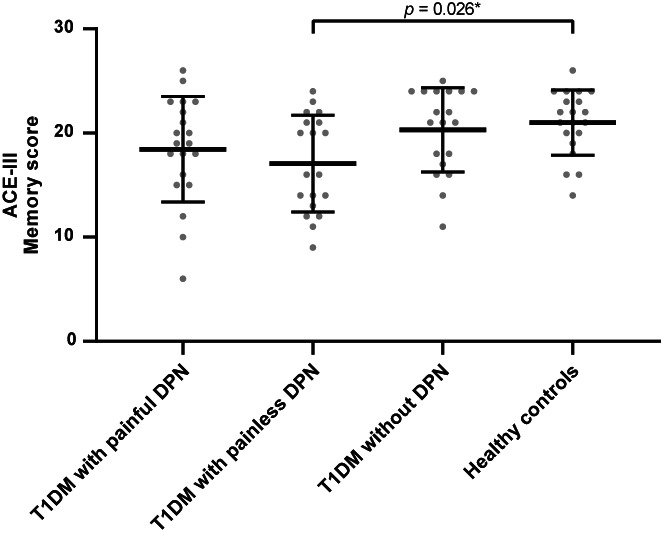
The memory score from ACE‐III for the group with T1DM and painful DPN, T1DM and painless DPN, T1DM without DPN, and healthy controls. The middle lines demonstrate the mean, and the error lines demonstrate the standard deviation. * represents a statistically significant difference between the indicated groups. ACE‐III, Addenbrooke's Cognitive Examination; DPN, diabetic peripheral neuropathy; T1DM, type 1 diabetes mellitus.

When comparing ACE‐III in the subgroups, an overall difference was observed for the memory domain (*p* = .022), as shown in Table 2. The post‐hoc analysis revealed a lower memory score in the participants with T1DM and painless DPN compared with healthy controls (*p* = .026), while no differences were observed between the other subgroups (all *p* > .096), see Figure 2. No differences were found for the total ACE‐III score or in the other domains (all *p* > .060).

The subgroups did not differ when comparing any parameters of the N‐back task including d’, RTs, or RTW (all *p* > .081), see Table 2.

### 3.4 Association between cognitive scores and clinical and peripheral parameters

Including all 58 T1DM participants, total and memory scores were not associated with age, diabetes duration, HbA1c, or NCS (all *p* > .058). However, there was a negative correlation between the language domain and age (*p* = .041). Furthermore, RTW during the 0‐back block was not associated with age, diabetes duration, HbA1c, or NCS (all *p* > .145). See Table 3 for more details.

**TABLE 3:** Correlations between clinical parameters and cognitive scores from Addenbrooke's examination III and the N‐back task for the T1DM participants (*n* = 58).

Clinical parameters	ACE‐III total score		Memory		Language		RTW (0‐back)	
Age (years)	*r* = −0.122^a^	*p* = .365	*r* = −0.065	*p* = .630	*r* = −0.272^a^	*p* = .041^b^	*r* = 0.185^a^	*p* = .169
Duration (years)	*r* = −0.154^a^	*p* = .251	*r* = −0.179	*p* = .179	*r* = −0.014^a^	*p* = .917	*r* = 0.195^a^	*p* = .145
HbA1c (mmol/mol)	*r* = −0.241^a^	*p* = .071	*r* = −0.250	*p* = .058	*r* = −0.080^a^	*p* = .555	*r* = 0.225^a^	*p* = .163
Sural amplitude (μV)	*r* = 0.107^a^	*p* = .426	*r* = 0.157	*p* = .240	*r* = 0.019^a^	*p* = .891	*r* = −0.111^a^	*p* = .409
Sural velocity (m/s)	*r* = 0.110^a^	*p* = .417	*r* = 0.174	*p* = .193	*r* = 0.002^a^	*p* = .990	*r* = −0.095^a^	*p* = .480

Abbreviations: HbA1c, haemoglobin A1c; RTW, weighted reaction times.

^a^ *n* = 57.

^b^ Represents a statistically significant association.

## 4 DISCUSSION

This study characterized cognitive function in individuals with T1DM compared to healthy controls and in well‐phenotyped T1DM subgroups with painful DPN, painless DPN and without DPN. Overall, participants with T1DM had significantly lower total, memory and language ACE‐III scores compared to healthy controls. Furthermore, the T1DM group had longer raw and accuracy‐weighted reaction times during the 0‐back task compared to healthy controls. No correlations were found between cognitive scores and clinical parameters in participants with T1DM. However, older age was associated with lower language scores. When comparing the subgroups, the participants with T1DM and painless DPN revealed a lower memory score than healthy controls.

The lower total ACE‐III score observed amongst the individuals with T1DM compared to the healthy controls coincides with our hypothesis and previous findings.^2^, ^4^ When applying a threshold of 82 points, 21% of the participants with T1DM met the criteria for cognitive impairment, while the prevalence was 5% for the healthy controls. Similar results were found in a study by Nunley and coworkers, where mild cognitive impairment was identified in 28% of individuals with T1DM and 5% of the healthy controls.^4^ Their T1DM group also included individuals with different types of diabetic complications. When comparing subgroups, we found no significant differences in the total ACE‐III score, suggesting that T1DM could be a general risk factor for cognitive impairment regardless of the presence of painful and painless DPN.

When examining the different cognitive domains, the individuals with T1DM had lower memory scores compared to healthy controls, suggesting that memory was affected in the T1DM participants. In the subgroups, the individuals with T1DM and painless DPN demonstrated lower memory scores compared to healthy controls, which could indicate that diabetic neuropathy is not only present in the peripheral nervous system but is also present centrally, and could thus have an influence on impaired memory function. This is in line with previous studies which suggest that peripheral nerve damage may serve as a risk factor for cognitive impairment.^4^, ^5^, ^11^ Nunley et al. observed that participants with T1DM and cognitive impairment were more likely to have DPN measured 5 years before cognitive assessment, while Ding et al. found a correlation between decreased sural nerve conduction velocity and lower cognitive scores, including memory score.^4^, ^5^ A recent systematic review also found cognitive domains like psychomotor speed and attention being affected in T1DM with DPN.^11^ However, our current study did not demonstrate associations between cognitive function and nerve conduction measurements. The inconformity between the studies might be attributed to the different sample sizes used across studies. Another explanation may be that NCS does not always detect early changes that occur due to nerve damage.^30^ Also, the cognitive changes might be attributed to other factors than peripheral nerve measures.

The group with T1DM and painless DPN had higher HbA1c levels than the T1DM group without DPN. This could have affected the observed difference in memory since elevated HbA1c has previously been associated with cognitive impairment.^2^, ^15^ Although, not significant this study demonstrated a trend toward an association between lower memory scores and higher HbA1c levels. Compared to the aforementioned studies, our study had a small sample size, which may have attributed to the borderline significant finding. Moreover, longer diabetes duration was observed in T1DM with painful DPN and painless DPN compared to T1DM without DPN. However, this was only borderline significant. Other studies found that cognitive deficit is affected by diabetes duration.^31^ Hence, these differences in diabetes duration might also contribute to lower memory scores in the DPN group.

Language scores were also mildly affected in the overall T1DM group compared to the healthy controls. However, the medians indicate limited effect size, and this difference is presumably not clinically relevant. Lower language scores were associated with higher age which might be one explanation for the observed results. Impairment of the language domain has not been a frequent finding in previous studies, which may be due to the choice of cognitive tests focusing on other domains. Nonetheless, Ding et al. also found lower language scores in T1DM compared to controls using two different cognitive tests.^5^

Additionally, individuals with T1DM exhibited longer reaction times during the 0‐back compared to the healthy controls, whereas the subgroups did not demonstrate significant differences. This indicates that psychomotor speed could be affected by T1DM, coinciding with previous studies which have also observed psychomotor slowing in individuals with T1DM.^3^, ^11^, ^32^, ^33^, ^34^, ^35^, ^36^, ^37^

Numerous studies have suggested that CNS is affected by T1DM,^38^, ^39^ including functional and structural alterations of brain areas involved in cognitive function such as memory and psychomotor speed domains.^40^, ^41^, ^42^ The functional and structural involvement of the CNS in T1DM may be one explanation for the observed altered cognitive function in this study. However, further studies are needed to confirm this.

Previous studies have suggested cognitive impairment in chronic pain. However, in our study, we did not find any difference in painful DPN group compared to the other subgroups. Pain‐related cognitive impairment has often been associated with the presence of depression and sleep disturbance.^14^ It was unfortunately not possible to evaluate depression scores in this study population, which is a major limitation of the current study.

### 4.1 Strengths and limitations

The main strength of the present study was the inclusion of three very well‐characterized diabetes groups with different disease characteristics and a group of healthy controls, which allowed us to investigate how T1DM, painful DPN and painless DPN impact cognitive function. Yet, this study was not without limitations. This study was part of a larger study, hence the primary sample size calculation was not based on cognitive changes but changes in the peripheral small nerve fibres. The relatively small sample size due to the explorative nature of the current cognitive study is one of the limitations. However, to our knowledge, this study is one of the first to investigate cognitive function in well‐phenotyped neuropathy groups and provide great relevance for future work, especially for the investigations at the subgroup level. ACE‐III is not validated for use in diabetes and functions more as a screening tool to detect mild cognitive impairment. But to our knowledge, no cognitive questionnaire validated in diabetes exists. Moreover, ACE‐III has shown to be sensitive to mild cognitive impairment, which is expected in diabetes. In comparison to HbA1c, continuous glucose monitoring would have been more suitable for assessing glucose regulation. It would also have been valuable to obtain information regarding the educational level, socioeconomic status, and previous hypoglycaemic episodes. Data were not adjusted for covariates that could influence cognitive function, such as age. However, this factor did not differ between groups as they were matched for age.

## 5 CONCLUSION

This is the first study to provide a comparison of cognitive function between T1DM with painful DPN, T1DM with painless DPN, T1DM without DPN and healthy controls. The study supports the notion of cognitive alterations in T1DM and indicates that cognitive function is altered in T1DM regardless of underlying neuropathic complications. The memory domain appears altered in T1DM, particularly in those with painless DPN. Due to the complexity of T1DM and its complications, it cannot be clearly concluded whether the observed cognitive impairment in individuals with T1DM is a result of nerve damage, microvascular disease, chronic pain, etc., or a combination of disease characteristics that may be linked to the development of DPN and cognitive impairment respectively. Individuals with painless DPN seem to be more likely to present with impaired memory. However, due to our small sample size, we cannot draw definite conclusions and larger longitudinal studies in well‐characterized T1DM groups with larger sample sizes are needed to confirm our results. This explorative study provides relevant insights about the cognitive changes in T1DM, regardless of underlying neuropathic complications. This study also indicates neuropathy as a potential contributing factor to the cognitive changes seen in individuals with T1DM. The findings of this study highlight the need for further research, especially in the area of T1DM and neuropathy, to better understand the mechanism underlying cognitive alterations in T1DM and to identify strategies for prevention and management of cognitive health in individuals with T1DM.

## AUTHOR CONTRIBUTIONS

**Suganthiya S. Croosu:** Conceptualization (equal); data curation (lead); formal analysis (supporting); investigation (lead); methodology (lead); project administration (lead); supervision (equal); validation (equal); visualization (equal); writing – original draft (supporting); writing – review and editing (equal). **Mimoza Gjela:** Formal analysis (lead); validation (equal); visualization (equal); writing – original draft (equal); writing – review and editing (supporting). **Johan Røikjer:** Conceptualization (equal); data curation (supporting); methodology (supporting); project administration (lead); writing – review and editing (equal). **Tine Maria Hansen:** Conceptualization (equal); methodology (supporting); supervision (equal); writing – review and editing (equal). **Carsten Dahl Mørch:** Conceptualization (equal); methodology (supporting); resources (supporting); writing – review and editing (equal). **Jens Brøndum Frøkjær:** Conceptualization (equal); funding acquisition (equal); methodology (supporting); resources (lead); supervision (equal); writing – review and editing (equal). **Niels Ejskjaer:** Conceptualization (equal); funding acquisition (equal); methodology (supporting); resources (lead); supervision (equal); writing – review and editing (equal).

## FUNDING INFORMATION

This research did not receive any specific grant from funding agencies in the public, commercial, or not‐for‐profit sectors.

## CONFLICT OF INTEREST STATEMENT

None of the authors have potential conflicts of interest to be disclosed. All authors have approved the final version of the article.

## Data Availability

Some or all data sets generated during and/or analysed during the current study are not publicly available but are available from the corresponding author upon reasonable request.
